# Supplementary material for: Analysis of the Contribution of Intrinsic Disorder in Shaping Potyvirus Genetic Diversity
Source: Viruses. 2022 Sep 3;14(9):1959. doi: 10.3390/v14091959 (PMC9504506; doi:10.3390/v14091959)
Supplement: Supplementary file 1 [file viruses-14-01959-s001.zip › Supplemental data Lafforgue.pdf]

Table S1. Mutations in coding sequences detected during experimental evolution in PVY 2015 1. and PVY 2017 2. or in TEV 2015 3.

1. Kutnjak et al. J Virol. 2015;89: 4760–4769. doi:10.1128/jvi.03685-14
2. Kutnjak et al. J Virol. 2017;91. doi:10.1128/jvi.00690-17
3. Cuevas et al. Mol Biol Evol. 2015;32: 1132–1147. doi:10.1093/molbev/msv028

Table S2. List PVY, TuMV and TEV isolates used to the natural diversity dataset.

Table S3. Adapted promotor score

from Radivojac P et al. Biophys J. 2007;92: 1439–1456. doi:10.1529/biophysj.106.094045

Figure S1. Variation of R^2, the coefficient referring to the correlation between percentage of mutations (S or NS) and protein length in the TEV genome, versus the mutations number. For a given number of mutations, 4 independent simulations were run.

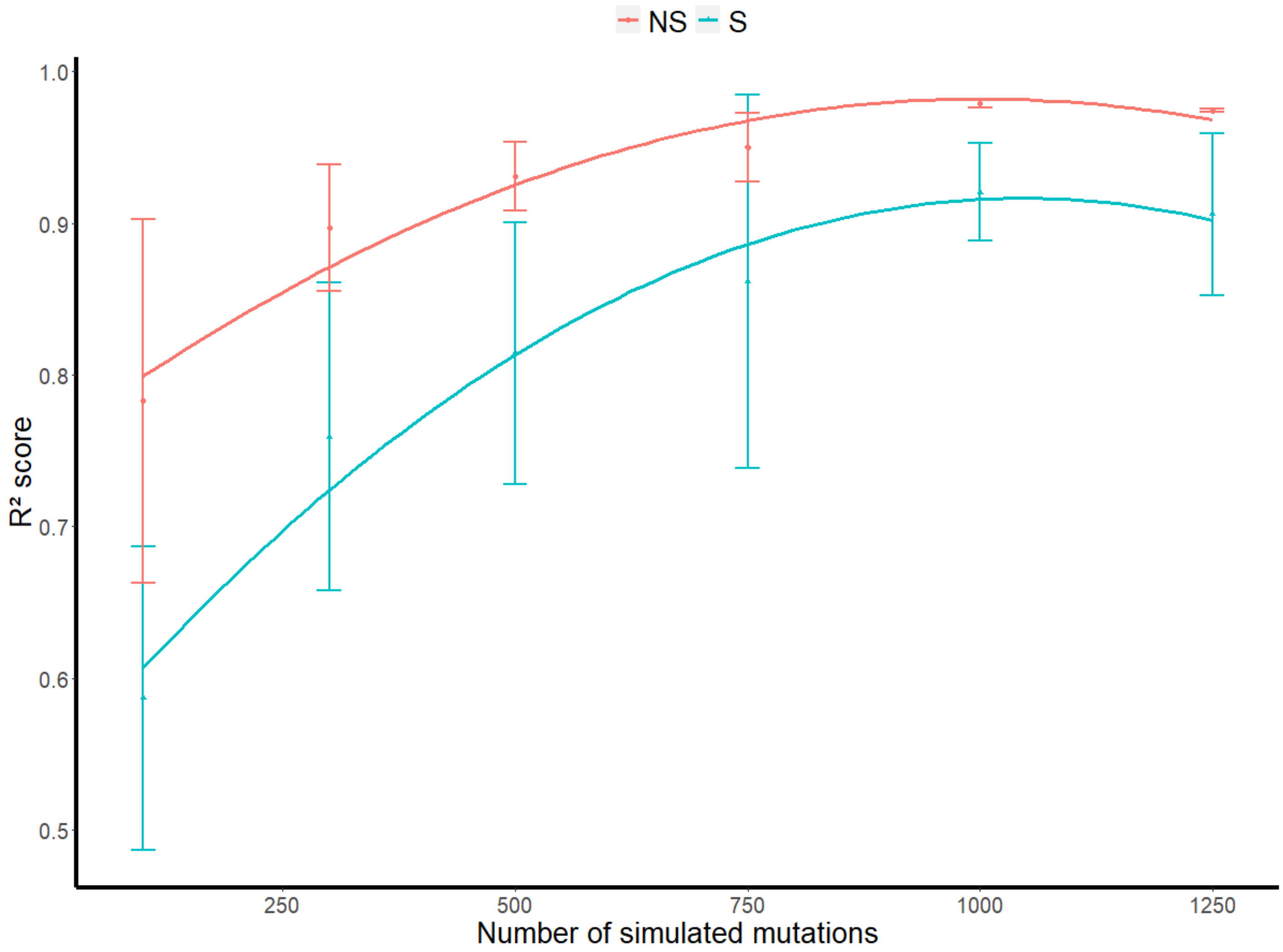

Figure S2. Mutation % in A. PVY proteins from experimental evolution (PVY 2015-2017), natural diversity (PVY_{ND}) and simulations. B. TuMV proteins natural diversity ($TuMV_{ND}$) and simulations. The proteins are sorted from the smallest to the largest, left to right: 6K1, 6K2, Nia-VPg, Nia-Pro, CP, P1, P3, HcPro, Nib, CI.

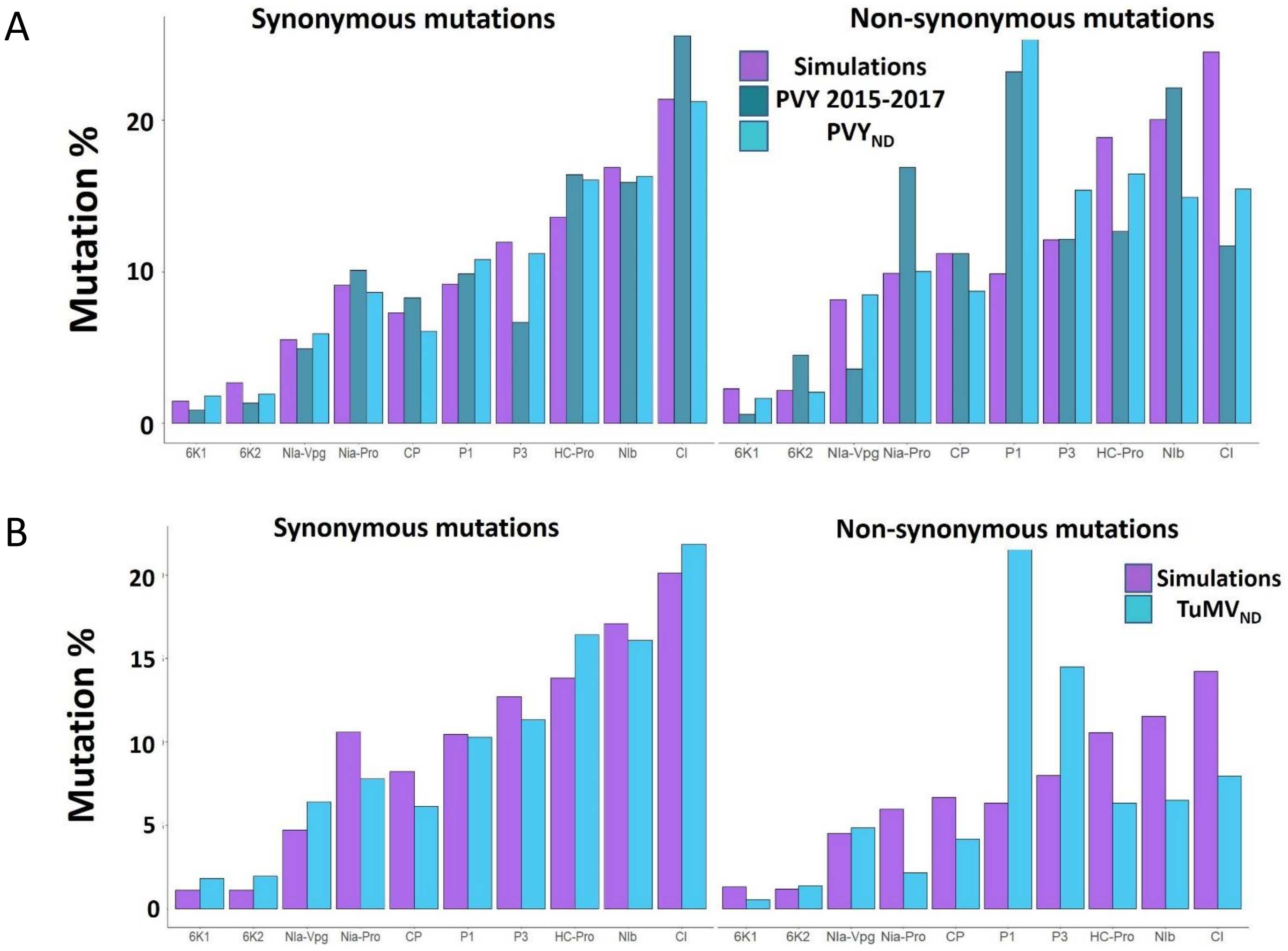

Figure S3. Ratio between the percentage of mutations (S or NS) present in IDRs and ORs for PVY (A), and TuMV (B). From left to right, data simulated (4 simulations), datasets from the experimental evolution (PVY 2015-2017) and natural biodiversity (PVY_{ND} or $TuMV_{ND}$).

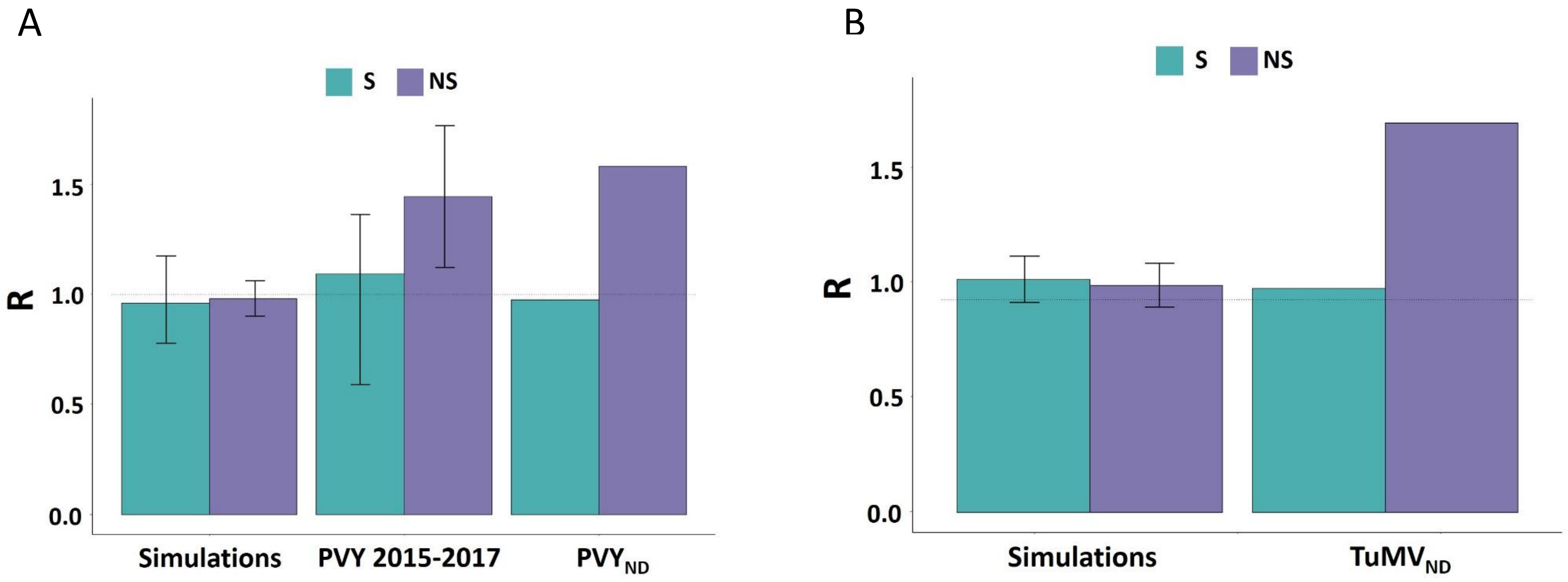

Figure S4. Mean codon volatility comparison between IDRs and ORs. A) Codon volatility obtained from TuMV natural (plain) and simulated data (shaded); B) Codon volatility obtained from PVY natural (plain) and simulated data (shaded); C) Codon volatility obtained from TEV natural (plain) and simulated data (shaded). Non-parametric Mann-Whitney U-test, p-value < 0.01.

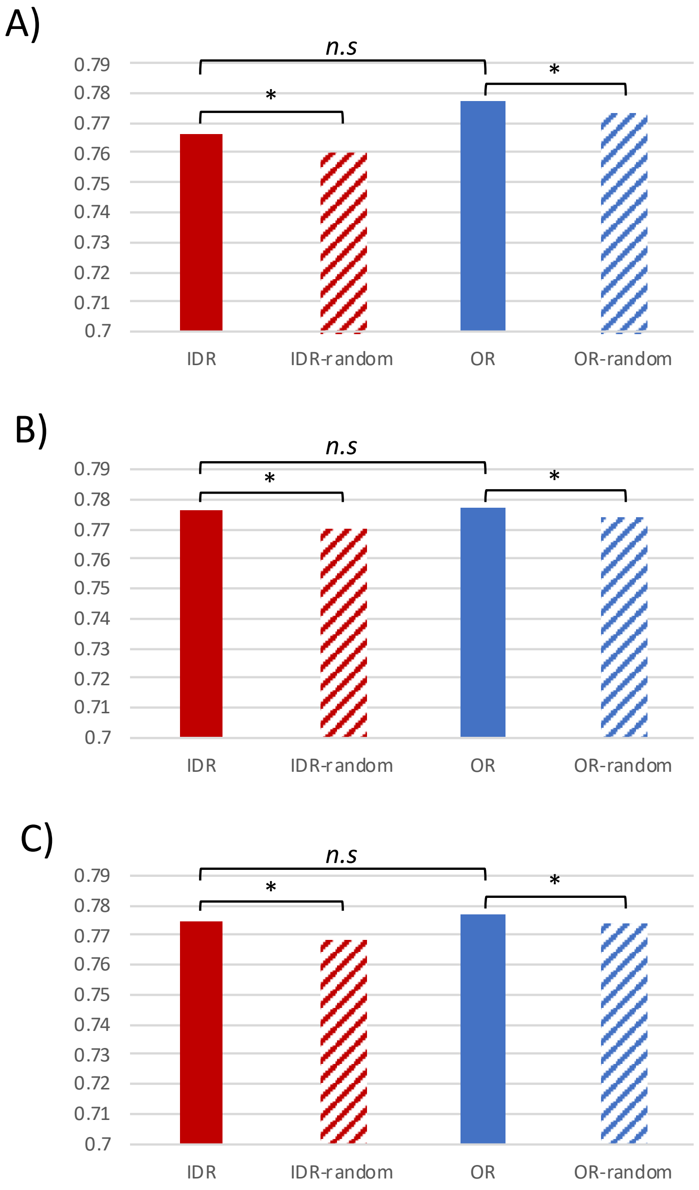

n.s : non significative

* : significative (p<0.05)
